# A Survey on the Relationship between Emotional Intelligence and Level of Depression and Anxiety among Women with Breast Cancer

**Published:** 2017-01-01

**Authors:** Nasrin Amirifard, Mehrdad Payandeh, Mehrnoush Aeinfar, Masoud Sadeghi, Edris Sadeghi, Somaye Ghafarpor

**Affiliations:** 1 Cancer Research Center, Kermanshah University of Medical Sciences, Kermanshah, Iran; 2 Medical Biology Research Center, Kermanshah University of Medical Sciences, Kermanshah, Iran; 3 Department of General Psychology, Kermanshah University of Medical Sciences, Kermanshah, Iran

**Keywords:** EQ, Anxiety, Depression, Breast cancer

## Abstract

**Background:** Emotional Intelligence (EQ) is positively associated with mental health and it can have a crucial role in mental disorder therapy by suitable coping mechanisms. The present study aimed to evaluate the relationship of EQ with anxiety and depression among the women with breast cancer.

**Subjects and Methods:** During 2013 and in a cross-sectional study, 98 breast cancer patients (14 to 21 years old) entered into the study. For data collection, the following instruments were the Bar-On EQ inventory, Beck Depression Inventory and Cattle Anxiety Inventory.

**Results:** There was an inverse relationship between anxiety with intrapersonal (p<0.01, r=0.39) and stress management (p<0.01, r=0.37) components and also between anxiety and total scores of EQ (p<0.05, r=0.22). There was an inverse significant association between depression and intrapersonal components (p<0.05, r=0.23), general mood (p<0.01, r=0.46) and adaptation (p<0.01, r=0.38) and also between depression and a total score of EQ (p<0.01, r=0.42).

**Conclusion:** The results of this present study confirmed the important role of EQ. Also, the results can be an inspiration for the future studies regarding the training of EQ skills in the treatment of mental disorder (anxiety and depression) among patients with breast cancer.

## Introduction

 Breast cancer is one of the most frequent malignancies among women of Iran and the world.^1^^,^^2^ Depression and anxiety are common in cancer patients and can be the results of illness, disease physiological factors or drug treatments.^3^ Depression and anxiety are also two of the most common reasons for alternative therapies in the community.^3^ The EuroQol Five Dimensions Questionnaire (EQ-5D) is a generic health-related quality of life (HRQoL) measure that is used for the purpose of economic evaluations of health interventions that consists of two parts: a self-classifier and a Visual Analogue Scale (EQ VAS). The self-classifier comprises five items relating to issues in the following domains: mobility, self-care, usual activities, pain/discomfort and anxiety/depression. Therefore, it has to be responsive to meaningful changes in health in the patient population under investigation.^4^^,^^5^ Emotional intelligence (EI) involves the perception, processing, regulation and management of emotions.^6^ The EI is one such personal characteristic and is increasingly made reference to in medicine, nursing and other health care professions.^7^ It is suggested that EI is important for effective practice, particularly with respect to delivering patient-centered care.^7^ Higher trait EI scores were also associated with better after-task recovery from stress experienced during the task.^8^ People with low emotional intelligence suffer from mental disorders, lack of empathy, anxiety, anger, weak defense mechanisms and have problems in administration of their emotions more than others.^9^ The main goal of the present study was the evaluation of the relationship between EQ with anxiety and depression.

## SUBJECTS AND METHODS

 This study was approved by the Ethics Committee of Kermanshah University of Medical Sciences (Ethical code: KUMS.REC.1394.499). Between Jun 2013 to Nov 2013 and in a cross-sectional study, 200 women with breast cancer referred to Breast Cancer Research Center, Imam Reza Hospital, Kermanshah, Iran. During the period, 105 patients met the inclusion criteria that were the patients with 14 to 21 years old. Seven patients had incomplete questionnaires and therefore they were excluded from the study. At last, the data of questionnaire for 98 patients were analyzed. For data collection, the following instruments were used:

- The Bar-On EQ inventory: This questionnaire is consisting of 15 factors and 5 main intrapersonal, adaptation, general mood, interpersonal and stress management components.^10^
- Beck Depression Inventory (BDI): It is a self-regulating questionnaire, 21-item version.^11^
- Cattle Anxiety Inventory: The Cattle inventory is a 40-item version of anxiety. For each question, maximum score 2 and minimum 0 is used. Scores can range from 0 to 80 and they are changed into (0-10). This test was a translated and validated Iranian version of Cattle's Inventory in 1988-1989.^12^

After obtaining the consent of the subjects and before completing the questionnaires, the aims of the study were responding to the participants and there were ensured that the information is confidential. The inventories were presented to the patients in two stages with good interval. After completing the questionnaires, the collected data were analyzed by SPSS version 19 and by Pearson correlation coefficient test.

## Results

Table 1 shows that there was an inverse relationship between anxiety with intrapersonal (p<0.01, r=0.39) and stress management (p<0.01, r=0.37) components that increasing anxiety, the intrapersonal and stress management scores were reduced. Also, there was an inverse significant association between anxiety and a total score of EQ (p<0.05, r=0.22) that reducing EQ, anxiety was increasing. As shown in Table 2, there was an inverse significant association between depression and intrapersonal components (p<0.05, r=0.23), general mood (p<0.01, r=0.46) and adaptation (p<0.01, r=0.38). Therefore, with increasing depression; intrapersonal intelligence, the general mood and adaptation were reduced. Also, there was an inverse significant association between depression and total score of EQ (p<0.01, r=0.42). It means that by the reduction of EQ, depression was increasing or vice versa. There is no significant relationship between depression with stress management and interpersonal components of EQ. Also, there was no significant relationship between age and education variables with anxiety and depression but there was a positive significant association between education and age with a total score of EQ.

## Discussion

 Breast cancer is a heterogeneous disease with distinct pathological and histological features.^13^ There is little in the literature exploring the inter-relationship of social support, prevalent symptoms and health-related quality of life (HRQoL) among breast cancer patients.^14^ Research and theory in this area suggest four factors that may be associated with anxiety and depression following breast cancer diagnosis: (Ι) demographic variables of age and educational level and degree of disease severity, (ΙΙ) two dispositional characteristics of individuals: optimism/pessimism and the tendency to monitor threat-relevant cues in stressful situations, (ΙΙΙ) the stress-response symptoms of avoidance and intrusive thoughts, and (ΙV) the use of engagement and disengagement coping strategies.^15^

**Table 1:** Pearson correlation coefficients to evaluate the relationship between anxiety and EQ among the patients with breast cancer

**EQ**	**Intrapersonal**	**Adaptation**	**General mood**	**Interpersonal**	**Stress management**	**Total score**
Anxiety	-0.39 **	-0.07	-0.09	-0.08	-0.37**	-0.22*

* **p**<0.05,

** **p**<0.01

**Table 2:** Pearson correlation coefficients to evaluate the relationship between depression and EQ among the patients with breast cancer

**EQ**	**Intrapersonal**	**Adaptation**	**General mood**	**Interpersonal**	**Stress management**	**Total score**
Depression	-0.23 **	-0.38**	-0.46**	-0.15	-0.11	-0.42**

* **p**<0.05,

** **p**<0.01

Depression and anxiety symptoms are relatively common among breast cancer patients.^16^ Emotional stress due to cancer diagnosis may unfavorably affect quality of life and the most common psychiatric problems in cancer patients are adaptation disorders and major depression, which may affect patient life quality, self-care, treatment compliance, and severity, progression and response to the treatment in the course of time.^17^ A study,^18^ showed that perceptions of relationship quality from women with breast cancer and their partners were negatively associated with partners' anxiety. Depression is seen in many cancer patients. It is common in the general population and in adults and children with cancer and frequently coexists with anxiety and pain.^19^ Slovacek et al.^20^ showed that subsist association between metastatic breast cancer, depression, and the low level of the global QoL. The results of the present study showed that there was a significant negative association between EQ with anxiety and depression among the patients with breast cancer. It was observed that there was a significant negative relation between anxiety and intrapersonal and stress management of EQ. There was a significant negative association between depression with intrapersonal and general mood components. Two studies,^20^^,^^21^ reported that monitored factors (gender, marital status and educational level) had no influence on the subjective health condition of the patients, as recorded by the EQ-5D scores but in our study, there was a positive significant association between education and age with a total score of EQ.

Extremera et al.^22^ showed that high Emotional Attention was positive and significantly related to high anxiety, depression, and to low levels of Role Emotional, Social Functioning, and Mental Health. However, high levels of emotional Clarity and Mood Repair were related to low levels of anxiety and depression, high Role Physical, Social Functioning, Mental Health, Vitality, and General Health. Although, many factors play an important role in mental disorder, including depression and anxiety, the results of the present study showed that EQ is a good predictor to suffer from depression and anxiety among the patients. In a general summary, it can be said that most of the researchers believe that if a person is emotionally potent, he can cope well with the life challenges including illness, social, family and economic issues. Thus, the patients with the ability to recognize, control and apply EQ capabilities have high social support, satisfaction and psychological health. As women have a crucial role in the family, emotional problems with the illness can play an important role in mental health threatening of the families of this group. EQ theory provided a new view regarding the primary prevention of mental disorder (anxiety, depression).

## CONCLUSION

 The results of the present study supported the important role of EQ. However, extensive research in this field is highly required. The results of the present study can be an inspiration for the future studies regarding the training of EQ skills in the treatment of mental disorder (anxiety and depression) among these patients.
